# Better than nothing? Patient-delivered partner therapy and partner notification for chlamydia: the views of Australian general practitioners

**DOI:** 10.1186/1471-2334-10-274

**Published:** 2010-09-20

**Authors:** Natasha L Pavlin, Rhian M Parker, Anna K Piggin, Carol A Hopkins, Meredith J Temple-Smith, Christopher K Fairley, Jane E Tomnay, Francis J Bowden, Darren B Russell, Jane S Hocking, Marian K Pitts, Marcus Y Chen

**Affiliations:** 1 Department of General Practice, The University of Melbourne, 200 Berkley Street, Carlton, Victoria, Australia; 2 Australian Primary Health Care Research Institute, Australian National University, Canberra, ACT, Australia; 3 Melbourne Sexual Health Centre, 580 Swanston Street, Carlton, Victoria, Australia; 4 Melbourne School of Population Health, The University of Melbourne, Carlton, Victoria, Australia; 5 Centre of Excellence in Rural Sexual Health, School of Rural Health, The University of Melbourne, 49 Graham Street, Shepparton, Victoria, Australia; 6 Australian National University and Canberra Sexual Health Centre, Garran, ACT, Australia; 7 Cairns Sexual Health Service, Cairns Base Hospital, Cairns, Queensland, Australia; 8 Key Centre for Women's Health in Society, The University of Melbourne, Carlton, Victoria, Australia; 9 Australian Research Centre in Sex, Health and Society, Latrobe University, 215 Franklin Street, Melbourne, Victoria, Australia

## Abstract

**Background:**

Genital chlamydia is the most commonly notified sexually transmissible infection (STI) in Australia and worldwide and can have serious reproductive health outcomes. Partner notification, testing and treatment are important facets of chlamydia control. Traditional methods of partner notification are not reaching enough partners to effectively control transmission of chlamydia. Patient-delivered partner therapy (PDPT) has been shown to improve the treatment of sexual partners. In Australia, General Practitioners (GPs) are responsible for the bulk of chlamydia testing, diagnosis, treatment and follow up. This study aimed to determine the views and practices of Australian general practitioners (GPs) in relation to partner notification and PDPT for chlamydia and explored GPs' perceptions of their patients' barriers to notifying partners of a chlamydia diagnosis.

**Methods:**

In-depth, semi-structured telephone interviews were conducted with 40 general practitioners (GPs) from rural, regional and urban Australia from November 2006 to March 2007. Topics covered: GPs' current practice and views about partner notification, perceived barriers and useful supports, previous use of and views regarding PDPT.

Transcripts were imported into NVivo7 and subjected to thematic analysis. Data saturation was reached after 32 interviews had been completed.

**Results:**

Perceived barriers to patients telling partners (patient referral) included: stigma; age and cultural background; casual or long-term relationship, ongoing relationship or not. Barriers to GPs undertaking partner notification (provider referral) included: lack of time and staff; lack of contact details; uncertainty about the legality of contacting partners and whether this constitutes breach of patient confidentiality; and feeling both personally uncomfortable and inadequately trained to contact someone who is not their patient. GPs were divided on the use of PDPT - many felt concerned that it is not best clinical practice but many also felt that it is better than nothing.

GPs identified the following factors which they considered would facilitate partner notification: clear clinical guidelines; a legal framework around partner notification; a formal chlamydia screening program; financial incentives; education and practical support for health professionals, and raising awareness of chlamydia in the community, in particular amongst young people.

**Conclusions:**

GPs reported some partners do not seek medical treatment even after they are notified of being a sexual contact of a patient with chlamydia. More routine use of PDPT may help address this issue however GPs in this study had negative attitudes to the use of PDPT. Appropriate guidelines and legislation may make the use of PDPT more acceptable to Australian GPs.

## Background

Genital chlamydia (*Chlamydia trachomatis*) infection is the most commonly notified sexually transmissible infection (STI) in Australia and one of the most prevalent worldwide [1]. Chlamydial infections can have serious reproductive health outcomes, particularly for women, and can result in tubal infertility, and ectopic pregnancy [2]. General practitioners (GPs) in Australia are ideally placed to conduct widespread chlamydia screening as nearly 90% of Australian women aged 15-24 years of age, the key risk group [2] visit a GP at least once each year [3]. As such GPs have been the focus for Australia's chlamydia screening efforts and are the providers most likely to be treating chlamydia. Australia's treatment guidelines for chlamydia include antibiotic treatment for the index case and their known partners and a repeat test for re-infection 3 months after treatment [4].

A cornerstone in the management of treatable STIs is the testing and treatment of sexual partners [5]. Effective partner management potentially prevents infection and its sequelae in partners and reduces the risk of re-infection in the index case.

There is concern that traditional methods of partner notification, such as patient or provider referral, are not reaching enough partners to effectively control the transmission of chlamydia [6-8]. Patient referral involves the person diagnosed with the STI contacting their partners themselves. Provider referral has traditionally meant public health staff, who are trained in partner notification, contacting partners on behalf of the patient. In recent years provider referral has also been used to describe health care providers - both medical and nursing - performing this function. Minimal evidence exists of the efficacy of this type of provider referral. There is limited availability of trained public health staff to perform partner notification in Australia and in practice only patients themselves and in some cases their doctors or nurses are active in partner notification for chlamydia. Various approaches have been tried around the world to improve notification and treatment of sexual partners. These include point of care counselling, internet and SMS based partner notification and patient-delivered partner therapy (PDPT) [5-7]. This involves the patient delivering antibiotics (e.g. single-dose azithromycin) to their sexual partner/s, without the partner attending a consultation with a health professional. Studies have shown PDPT to be as effective as, and in some cases more effective, than patient referral in both the proportion of sexual partners treated, and in reducing re-infection rates in index women [9-15]. PDPT is currently used in Sweden and parts of the USA [8,15], and has been recommended as an option for the management of chlamydia in draft guidance from the U.K. National Institute for Health and Clinical Excellence [15]. PDPT provides flexibility for treatment of partners who are unwilling or unable to attend a consultation with a health professional [6]. However, PDPT is a controversial practice as it involves a health professional providing medication for an individual that they have not met, nor clinically evaluated [15].

In Australia, research suggests GPs want guidance and support for partner notification and there is currently no specific legislation or guidelines supporting the use of PDPT [16]. Aside from legal considerations, clinicians contemplating PDPT also face potential medico-legal and ethical issues stemming from the treatment of individuals who are not "their" patients. Few published data exist on clinicians' use of and views on PDPT from countries where it is not specifically supported nor seen as normal clinical practice. This study aimed to determine the views and practices of Australian general practitioners (GPs) on partner notification, particularly in relation to PDPT and explored GPs' perceptions of their patients' barriers to notifying partners of a chlamydia diagnosis.

## Methods

Ethics approval was obtained from University of Melbourne Human Research Ethics Committee. All participants gave informed consent. In-depth, semi-structured telephone interviews were conducted with 40 GPs from November 2006 to March 2007.

GPs from Queensland, Victoria and the ACT (n = 9826 ) were initially identified by postcode from the AMPCo database [17]. GPs from Queensland and Victoria were then divided into rural and urban areas. A statistician randomised each group. GPs known to work in Indigenous Health were also approached via telephone listings in the White Pages Telephone Directory. GPs with experience in working with Indigenous Australians were oversampled to ensure their views were represented in the study.

To be eligible to participate GPs must have diagnosed at least one case of chlamydia in the past year.

Each week 15 rural and 15 urban GPs from Victoria and Queensland and 7 from the Australian Capital Territory (ACT) were contacted by mail or fax and followed up with a phone call until 40 GPs had been interviewed. Over an 11 week period there were 7 mail outs; 59 letters sent to rural Victoria; 59 to urban Victoria; 65 to rural Queensland; 60 to urban Queensland and 43 letters sent to GPs in the ACT. Forty letters were sent to GPs currently working in Indigenous health in rural Victoria, urban Victoria and rural Queensland.

Topics covered in the interviews included GPs' current practice and views about partner notification, barriers they experience and supports they need to more effectively implement the partner notification process for patients with chlamydia. Specific questions were asked about previous use of and views regarding PDPT. The interview schedule was piloted with two GPs and amended following review by authors RP and NP. NP conducted all interviews; she is a practicing GP and was able to relate to the participants' concerns and environment, and to probe their responses appropriately. NP was supervised by RP who has extensive experience conducting interviews on sensitive topics and acknowledged skills in qualitative research. RP regularly listened to interview recordings and provided feedback to NP.

Interviews took 30-40 minutes, were audio-taped, transcribed, imported into NVivo7 as a Word document and subjected to thematic analysis. [18]. To avoid bias, both NP and RP read all transcripts and developed a coding framework. Emerging themes were reviewed as the analysis progressed, and made use of the different perspectives of NP (GP researcher) and RP (sociologist). Data saturation was reached when no new themes emerged after the initial 32 interviews were analysed, although a further 8 were completed and analysed.

## Results

The 40 GPs interviewed work predominately in private general practice although as mentioned previously doctors working in Aboriginal Medical Services were deliberately oversampled. Table 1 describes the demographics of the GPs interviewed for this study.

**Table 1:** Demographics of GPs interviewed

Total GPs Interviewed	Private General Practice	Aboriginal Medical Service	University Health Service	Australian Defence Force	Occupational Medicine	After Hours Deputising Service
n = 40	26	8	2	2	1	1
			
Total GPs Interviewed	Australian Capital Territory	Victoria	Queensland			
			
n = 40	4	24	12			
			
Total GPs Interviewed	Urban Areas	Rural and Regional Areas				
				
n = 40	21	19				
				
Total GPs Interviewed	Female	Male				
				
n = 40	19	21				
				
Total GPs Interviewed	Australian Trained Medical Graduates	International Medical Graduates				
				
n = 40	29	11				

Communication difficulties (in terms of English language skills) were evident in five interviews. Twenty-three interviewees worked full-time in General Practice (defined as >30 hours per week) and 17 worked part-time ( < 30 hours per week). Twenty-five GPs reported no experience with Indigenous patients, five reported some experience with Indigenous patients and ten had very significant experience with Indigenous patients. All denied having a particular interest in sexual health.

### Current methods of partner notification

Various methods of partner notification were used by the GPs. A majority viewed partner notification as the patient's responsibility (patient referral), a finding consistent with other Australian studies [16,19,20]. Provider referral was undertaken by a small number of GPs. Many GPs expressed confusion about the 'correct' protocol for partner notification, and uncertainty about the roles and responsibilities of themselves, their patients and the public health authorities.

*...is it the GP's role to contact all their partners, or is it the DHS *[Department of Human Services]*, or who? (Dr RE 185)*

### Barriers to partner notification and treatment

Perceived barriers to patients telling partners included: the stigma of having an STI; patient factors such as age and cultural background; and relationship factors such as whether it is a casual or long-term relationship, and whether the relationship is ongoing or has ended.

...in terms of getting people to make sure that other relationships are notified... I think the conflict there is that for young people sometimes it's very traumatic to - to disconnect from an intense and intimate relationship. And having completed the disconnection, I don't think they actually want to revisit it on any level. (Dr DC 246)

And so... they might've had a number of boyfriends over a reasonably short period of time. And you sort of try to say, well, it could be any of those guys, so maybe you should actually inform them all. But that's very threatening... and I'm sure they don't do it. (Dr KN 256)

Barriers to the GPs themselves undertaking partner notification (provider referral) included: lack of time and staff to undertake the work; logistical difficulties such as lack of contact details; uncertainty about the legality of contacting partners and whether this constitutes breach of patient confidentiality; and feeling both personally uncomfortable and inadequately trained to contact someone who is not their patient.

Well, resource number one, time. Number two, which staff member would do it, so you'd have to allocate a person to it, and that is all work for free. (Dr DL ATSI 348)

I think it's, you know, hard to be on the end of the phone and going, "Hello, you don't know me from a bar of soap but I think we need to test you for chlamydia". You know that conversation is always going to be a hard one and I think that is a big barrier. (Dr GY 371)

Many GPs felt that a particular barrier to effective treatment of partners is that, even once notification has occurred, many partners are unwilling or unable to attend a consultation with a health professional.

I guess the main barrier we would see is that people won't come in. We do have an urgent recall as I say for more than six months, twelve months and they just won't come in, so whether they've got a suspicion of what it is, or they just haven't sort of formed a rapport with the practice, um, that'd be our main thing... (Dr EC ATSI 306)

*Probably the other thing is gender related because males are reluctant to come to the doctors in general, and the partners - the patients who are screening are mostly females, and males don't come to the doctor, so probably that's a barrier, the gender itself*. (*Dr EP 241*)

The - you know, the amount of trouble you have to go to contact some people is - is astounding. And even when you get on to them and tell them that they have to come, they still don't come. (Dr KN 512)

### Facilitators for better partner notification and treatment

GPs identified the following factors which they considered would facilitate partner notification: clear clinical guidelines; a legal framework around partner notification; a formal chlamydia screening program; financial incentives; education and practical support for health professionals, and raising awareness of chlamydia in the community, in particular amongst young people.

I think it would be...more a clear system change. So, some sort of... clear guidelines for a GP to follow, and, you know, with the - the legal or government backup that we're sort of obliged to do this thing. And, you know, therefore involve the patient and everything in a way that's a bit less ambiguous than it is now. (Dr EA ATSI 721)

*I mean the public awareness of it - if there was a national campaign which would run, would obviously be a lot better. So that might be...helpful I guess in some respects. But the awareness of it would be increased, so that... perhaps people would then have a better baseline knowledge in terms of...contact tracing...and the importance of it, and ah, it's necessary - necessary nature of it*. (Dr TM 447)

### Views on PDPT

Slightly fewer than half of the GPs expressed positive views about PDPT and slightly more than half expressed negative views about PDPT. Of those who expressed negative views it appeared that a small minority felt strongly against PDPT and the remainder had more moderate concerns. A small number of the interviewed GPs did not have any particular views on PDPT.

### Positive attitudes to PDPT

Those with positive attitudes towards PDPT mostly expressed the viewpoint that while the ideal situation would be to clinically evaluate the partner, PDPT is preferable to no treatment at all. They considered that the benefits outweighed the risks.

... it's probably best to see them anyway... I mean, for counselling. But if you suspect that they will never come back, it's probably a better result for population health rather than not give anything. (Dr EP 190)

*But if you've got a situation where that's your only option for treatment them, then ah, it's better than nothing, I would say*. [In] *the scheme of things the ideal care would be to have them come in, and talk to them about all their sexual stuff, and make sure there are no other things that you need to treat or deal with. But if you've got a partner who's just not going to come in, and the only way you're going to get them treated is if the girlfriend takes - takes them home and gives them *[the medication] *- I mean, you know. It's probably better than not treating them at all. (DR HR ATSI 330)*

Some GPs considered PDPT, including the fact that they were not actually testing the partner for chlamydia, to be acceptable as usual practice would be to treat such patients empirically due to known exposure to a case.

In this scenario it's easier because we know that he has to be treated, so - and we know that - and you would treat him anyway. We wouldn't do swabs. We would treat him anyway because he's - he's a possible contact. So I think it's all right to give a script without seeing someone if - if that's - would be the end result anyway. (Dr EP 183)

Several GPs considered that PDPT had evidence-based benefits.

I've actually - um, about a year ago I looked up sort of one of these evidence-based, ah, web pages and I saw that there is some evidence to say that that is a good idea, and we would commonly do it. (Dr EC ATSI 277)

### Negative attitudes to PDPT

Clinical, medicolegal and ethical concerns regarding the use of PDPT were expressed by some GPs.

### Clinical concerns

The most commonly expressed concern was that PDPT did not represent best clinical practice. Specific concerns included: the lack of opportunity to undertake a complete history and clinical examination, in particular to assess for contraindications to medication such as allergies or drug-drug interactions; lack of diagnostic testing before, and follow-up testing after treatment; lack of investigation for complications of chlamydial infection or concurrent STIs; missed opportunity for information sharing between the health professional and the partner; and lack of further tracing of the partner's other sexual contacts.

*Well, I don't think that would be looked at as a reasonable option by the College *[Royal Australian College of General Practitioners] *or standards or peers. (Dr EG 288)*

There are medical issues with that - can you be sure of what their allergies are? Can you be sure that they are not pregnant or not taking some sort of other medication that will interfere with the normal treatment? Definitely it deprives them of a follow up, in that most people - all people with chlamydia should be tested two weeks later to confirm clearance of the infection. That isn't going to happen if you prescribe by proxy. The other last thing that is of significance is that if somebody has chlamydia that greatly enhances their risk of acquiring another sexually transmitted disease at the same time. Treating the chlamydia alone is arguably inadequate treatment for the other partner. (Dr BK 192)

Many GPs were concerned that PDPT might not be effective. A particular concern was whether the doctor could be sure that the partner has been notified and given the script, and whether the script is filled and the medication goes to the person it was intended for.

### Medicolegal and ethical concerns

Some GPs had specific concerns about the medicolegal implications of PDPT.

And can you imagine standing in the Coroner's Court saying, "Yeah, well, I hadn't even seen them, hadn't even met them before, and um, you know, I wrote a script for them, or wrote a script for my patient knowing full well that they were going to give it to somebody else." I mean, it's - it's illegal. (Dr MS 227)

Others were concerned that patients may use the medication to treat their partners without the partner's full understanding, and that this would not meet the ethical and legal requirement of informed consent for treatment.

I think the medico-legal issue there is probably the biggest one. If they're on some kind of concurrent medication or have an allergy - they need to know why they're taking it. I could foresee a scenario where it gets slipped into their, um, Vegemite toast in the morning...(Dr TM 365)

*And I think it's probably important to make sure that the information they're getting is the correct information, 'cause the partner may *[say]*, you know, "I just need you to take this 'cause I've got thrush and we need to treat the thrush," and they go, "Oh yeah, okay, that's fine." (Dr GY 344)*

## Discussion

This is one of the first qualitative studies to investigate Australian GPs' attitudes towards PDPT as an approach to treating partners of patients with chlamydia. Both positive and negative views were expressed by GPs. A barrier to effective management of partners as identified by many of the GPs is that some partners are unwilling or unable to seek timely medical treatment even after they are notified of being a sexual contact of a patient with chlamydia. This finding is significant, as it identifies a need that could be met by the use of PDPT, which provides a flexible alternative for treatment of partners who may otherwise be left untreated. Guidelines and legislation are required in Australia to support GPs in using PDPT where appropriate.

Through the interviews with the GPs, it became clear that there is no uniform approach to partner notification for chlamydia in Australia, a finding reported elsewhere [16,19]. While patient referral was the most commonly reported method used, provider referral was used by a smaller number of GPs. GPs felt they would be better able to facilitate partner notification if they received clarification about best practice, and about the roles and responsibilities of patients, GPs and the public health authorities. Education, tools and practical support for GPs were also identified as possible facilitators for partner notification in this study, and in other recent Australian studies [20,21]. Online services that allow patients to email or text message partners have also shown promise and could assist both GPs and patients with partner notification [6,21].

GPs in this study expressed negative views about PDPT. This is in contrast to similar surveys conducted in the U.K. and the U.S., in which a majority of doctors interviewed expressed positive views about PDPT, and/or a willingness to use it [6,8,22]. This difference may be related to the nature of the doctors included in the samples - in many of the overseas studies, the doctors sampled included gynaecologists, genitourinary physicians, or doctors with a particular interest in family planning and sexual health. In addition, PDPT is legal in some jurisdictions within the U.S. [23], and its use is encouraged, so doctors there are likely to be more comfortable about using PDPT.

Another important factor, which may explain this difference, is the context in which the use of PDPT is proposed. The concern most commonly expressed about PDPT by GPs in this study was that PDPT did not represent best practice. The GPs were clearly concerned about providing medication for someone whom they had never met, let alone clinically evaluated with a thorough history and examination. This is understandable as PDPT does represent a significant departure from the traditional doctor-patient relationship, particularly in a setting such as Australia with a tradition of strict regulation of prescribed medications. However, PDPT as it is used overseas is not intended to replace best practice, but to be available as an option to treat partners who would otherwise not be treated at all. For example, Californian guidelines for the use of PDPT state that its use should be restricted to "*those with partners who are unable or unlikely to seek timely clinical services*" [24]. Similarly, in this study, those GPs who supported PDPT recognised that it was not best practice, but that it could be a useful compromise in situations where the alternative would be no treatment at all.

In addition to clarification about the situations in which PDPT is appropriate, other practical concerns expressed by the GPs in this study would also need to be addressed before it is seen as a legitimate treatment option. Many GPs felt that PDPT may not be safe, particularly because the patient would not be assessed for the possibility of allergic reactions to the medication. However, azithromycin, the drug usually prescribed in PDPT, is generally a well-tolerated and safe antibiotic, with a low incidence of allergic reactions [15]. Although a number of GPs considered that PDPT may not be very effective, some studies have found that it is equally or more effective than patient referral partner notification [9-15], the method currently employed by the majority of GPs in Australia.

GPs were concerned that PDPT may result in incomplete care for the partner, since they are not evaluated for complications of infection (such as pelvic inflammatory disease in women), or for concurrent STIs. While the likelihood of such complications and concurrent infections depends on the population of interest, a recent Australian study has concluded that the incidence of these complications and concurrent infections in heterosexual partners is low enough to recommend the use of PDPT [25]. Further studies should be done to confirm these findings, and to help clarify the populations in which PDPT could be safely used.

Another concern was the lack of opportunity to trace the partner's additional sexual contacts. A recent audit conducted in Scotland found that the use of PDPT meant that 22-28% fewer additional cases of chlamydia were found than when partners were clinically evaluated and secondary sexual contacts traced [26].

It should be noted that if the alternative to PDPT is not informing of partners at all, this also prevents evaluation of the sexual partner for complications and concurrent infections, and secondary contact tracing. Therefore, some may consider that it is preferable to use PDPT in these settings, as at least it has the benefit of empirically treating the chlamydial infection of the sexual partner.

A strength of this study is the inclusion of GPs across a broad range of backgrounds and locations across Australia. As this was a preliminary study, and the interviews had a broad scope, some issues identified would benefit from further in-depth study. Future research on the attitudes of GPs towards PDPT, with more probing questions and scenario-based discussion, would allow for deeper understanding of this complex issue. In addition, an important area for further research would be the views of patients themselves on the use of PDPT [6].

## Conclusions

This small qualitative study of Australian GPs found that at present more GPs have negative rather than positive views about PDPT for the treatment of sexual partners of patients with chlamydia. Many of the concerns expressed by the GPs are likely to be ameliorated by the provision of further information about the appropriate use of PDPT and its safety and effectiveness. Ultimately, guidelines, decision support tools and legislative changes that specifically support PDPT are needed, before practitioners feel comfortable with the use of PDPT.

## Competing interests

The authors declare that they have no competing interests.

## Authors' contributions

NP contributed to study and interview schedule design, conducted, recorded and analysed all interviews, contributed to drafting and review of the manuscript. AP prepared the first draft of the manuscript. RP contributed to the design of the study, assisted with analysing interviews and developing themes. CF conceived the idea for the study, was the principle investigator on the funding grant and contributed to the design of the study and review of the manuscript. MTS and JT contributed to the design of the study and themes explored. FB, DR, JH and MP contributed to study design and reviewed the manuscript. MC contributed to the design of the study, concepts and themes explored, reviewed the manuscript and oversaw the project. All authors have contributed to checking of the manuscript and approval of the final version.

## Pre-publication history

The pre-publication history for this paper can be accessed here:

http://www.biomedcentral.com/1471-2334/10/274/prepub
